# Testing the efficacy of a minimal-guidance online self-help intervention for alcohol misuse in Estonia: study protocol of a randomized controlled trial

**DOI:** 10.1186/s12889-020-08791-6

**Published:** 2020-05-27

**Authors:** Esta Kaal, Michael P. Schaub, Andreas Wenger, Triin Ülesoo, Matthijs Blankers, Severin Haug, David D. Ebert, Heleen Riper, Matthew Keough, Helen Noormets, Karin Kilp

**Affiliations:** 1 Estonian National Institute for Health Development, Tallinn, Estonia; 2 Tallinn University|, Tallinn, Estonia; 3 Swiss Research Institute for Public Health and Addiction, University of Zurich, Zurich, Switzerland; 4 Department of Research, Arkin Mental Health Care, Amsterdam, The Netherlands; 5 Academic Medical Centre, Department of Psychiatry, University of Amsterdam, Amsterdam, The Netherlands; 6 Department of Clinical Psychology, Vrije Universiteit Amsterdam, Amsterdam, The Netherlands; 7 GGZ inGeest, Research and Innovation, Amsterdam, The Netherlands; 8 Department of Psychology, York University, Toronto, Ontario Canada

**Keywords:** Alcohol misuse, Cognitive behavioural therapy, Motivational interviewing, Online, Self-help

## Abstract

**Background:**

Despite an initial steep decrease in alcohol misuse among Estonians through structural intervention means and the scaling up of alcohol counselling in the mid-2000’s, most of the country’s alcohol misuse indicators remain clearly higher than European averages. Consequently, an online self-help program was launched as part of an initial behavioral intervention initiative to foster progress in alcohol prevention on a population level.

**Methods:**

A two-arm randomized controlled trial (RCT) has been designed to compare the efficacy of a culturally-adapted minimal-guidance online self-help program, the 8-week “Selge” online program against a control condition that consists of a self-administered test of alcohol use and advice regarding usual treatment in Estonia. A target sample of 600 individuals will be recruited and randomly assigned to either condition. The program will contain 10 modules based on principles of cognitive behavioural therapy (CBT) and motivational interviewing (MI). Participants in the control group will have access to the full treatment after they complete their final follow-up assessment. The primary outcome will be change in the Alcohol Use Disorders Identification Test (AUDIT) score between the 6-month follow-up and baseline assessments. Secondary outcomes will include the number of standard drinks consumed and alcohol-free days, drinking motives and motivation for change, as well as changes in mental health. Assessments will be completed at baseline, at the end of treatment, and at 6 months follow-up. Data analysis will follow the intention-to-treat principle and employ (generalised) linear mixed models.

**Discussion:**

The *“Selge” program* is the first and only internet program for the intervention of alcohol misuse in Estonia. If proven effective, it will foster progress in the intervention of alcohol misuse in the Estonian population and be implemented as a standard program amidst the continuum of intervention and care.

**Trial registration:**

Current Controlled Trials ISRCTN48753339 registered 04/06/2019 retrospectively.

## Background

Misuse of alcohol is a worldwide problem that has numerous negative consequences for public health. The World Health Organization (WHO) sees 6 l of pure alcohol consumed per capita per year as significant damage to public health [1]. Relative to 2007, when the consumption of alcohol by Estonian residents reached a peak of 14.8 l of pure alcohol per adult (15-years-old and older) per year, by 2015 consumption had decreased by more than 4 l, though it remained at the level of roughly 10 l through 2016 and 2017 [2]. Moreover, the prevalence of heavy episodic drinking among people age 15–24 is still higher than the European Union average and the alcohol-attributable contribution to overall mortality, as a fraction, in Estonian adults is almost four times the European average [3].

As a consequence, the National Institute for Health Development and the Ministry of Social Affairs of Estonia compiled the so-called *Green Paper on Estonian Alcohol Policy* to reduce alcohol abuse and harm in 2014. This was followed by various initiatives that included the program “More Sober and Healthier Estonia”, funded by the European Social Fund. The aims of this program are to a) improve the availability and quality of services needed to prevent and treat alcohol abuse; and b) to raise public awareness about the risks of alcohol consumption and existing support services. In the area of health services, the program designates action in two directions: 1) early detection of alcohol abuse by family physicians; and 2) treatment of alcohol abuse by medical specialists. Over the period 2014–2018, 22 health centres provided early detection and an Alcohol Disorder Patient Treatment Guideline was compiled, which also shows patient pathways within the healthcare system. By 2018, nine facilities had started providing the treatment quickly and effectively [4].

Despite this progress, there is still a long way to go. The recent survey (2018) on health behaviour shows that 14,7% of Estonian adults consume alcohol at a health threatening/ damaging alcohol level, while only 1,9% of respondents declared having been treated for alcohol abuse [5].

In addition to the early intervention and treatment scale up, the National Institute for Health Development (NIHD) has been co-ordinating a public awareness media campaign for over 10 years. In 2017, a campaign was initiated aiming to help risky drinkers to reduce their alcohol consumption, within the programme “Sober and Healthier Estonia”, and thereby avoid the harmful consequences of their drinking patterns. The campaign’s call to action directed people to conduct the AUDIT test [6] on the alkoinfo.ee webpage. Depending on their test score, respondents were provided with recommendations and links to further reading. After the campaign, 20% of the adult population and 30% among the primary target group (people ages 25–45 who consume alcohol above a low-risk level) were aware that they could self-evaluate their personal alcohol consumption using the AUDIT test. During this 2017 campaign, 70,324 visitors visited the website www.alkoinfo.ee where information on all stages of changing drinking behaviour is available, while 60,842 completed the AUDIT test on the alkoinfo.ee website [7]. Thus, the campaign spurred some action by 6,5 and 5.7% of the Estonian population over 18 years old, respectively. To expand this success and more effectively decrease alcohol misuse over the internet, the *“Selge” program* (the word“selge” has the double meaning of being clean and sober in Estonian), a minimal-guidance self-help program adapted from the “takecareofyou” program developed in Switzerland [8, 9] was developed in 2018.

According to a recent individualized-participant data meta-analysis, participants receiving internet interventions based on personalised (normative) feedback are significantly less likely to maintain low-risk drinking patterns at follow-up than those in extended internet programs based on integrated therapeutic principles like Cognitive Behavioral Therapy (CBT) and Motivational Interviewing (MI) [10]. Moreover, guided internet interventions exhibited greater effects on drinking patterns than unguided interventions [10]. However, only one study has directly compared a brief online intervention conceptualised as PNF (Personalized Normative Feedback) and a booklet with an unguided online self-help intervention. Although unguided, the latter was very intense, containing 56 daily sessions plus six weekly follow-up sessions, and involved alcohol misusers from the general population [11]. Although only 8 % completed all sessions in the intensive intervention group, there was a superior reduction in alcohol use in this group than in the PNF/booklet group over the medium term (6 months follow-up).

The goals of the currently-proposed study are to evaluate the efficacy of the adapted *“Selge” program*, in terms of reducing alcohol misuse in Estonians, and to assess its effects on treatment referrals. The intervention is adapted from the integrated alcohol misuse and depression symptoms treatment developed by collaborators [12] and consist of 10 modules based on MI and CBT. We expect that the *„Selge* “*program* will result in greater reductions in alcohol misuse than the control intervention, which will consist of a self test for alcohol abuse and routine advice regarding treatment.

## Methods

### Study design

The minimal-guidance Web-based self-help *“Selge” program* will be evaluated within a two-arm non-blinded randomized clinical trial (RCT). Eligible individuals will be randomized to either the online program or the control group (described below). Assessments will occur before randomization (T0; baseline), at 8 weeks (T1; treatment end), and at 6 months (T2; 4-month post-treatment follow-up). We have chosen these three measurement dates to distinguish intervention effects from possible follow-up effects. The control condition website will be accessible to both study conditions, and the actual use of treatment from usual resources assessed at T1 and T2. Participants will be made aware to which treatment arm they have been assigned after their baseline measurement. Participants allocated to the control condition will be provided with access to the full online program after they complete their final follow-up assessment.

### Data collection

Recruitment of study participants started on March 13th, 2019 and data collection will be continued through October 2019. Our aim is to recruit alcohol misusers from the Estonian general population through various means. Individuals are being recruited through several means across the country, including ad banners in web media platforms (Facebook, Instagram, Google search) and printed flyers in co-operation with general practitioners. A research institution press release was published by several local and one countrywide newspaper, and reported by one radio channel. Ad banners and publications invited people who would like to decrease their level of alcohol use to visit the research institute homepage for more information.

### Inclusion and exclusion criteria

To strengthen external validity, we applied very few inclusion/exclusion criteria. The main target group is Estonians with at least moderate misuse of alcohol. Inclusion criteria for participation are: 1) a minimum age of 18 years; 2) alcohol misuse, according to the Alcohol Use Disorder Identification Test (AUDIT score of ≥8); 3) at least weekly access to the internet; and 4) literacy in the Estonian language. Exclusion criteria are: 1) use of other psycho-social or pharmacological treatments to reduce the consumption of alcohol, opioids, stimulants or cannabis at baseline; 2) use of opioids, stimulants or cannabis more than four times over the previous 30 days; 3) previous treatment for cardiovascular problems; 4) pregnancy or breast feeding; and 5) suicidal ideations or plans within the last couple of years. Applicants who do not fulfill the criteria are informed that they do not fit the study but can still use the tool.

### Informed consent procedure

**Fig. 1:**
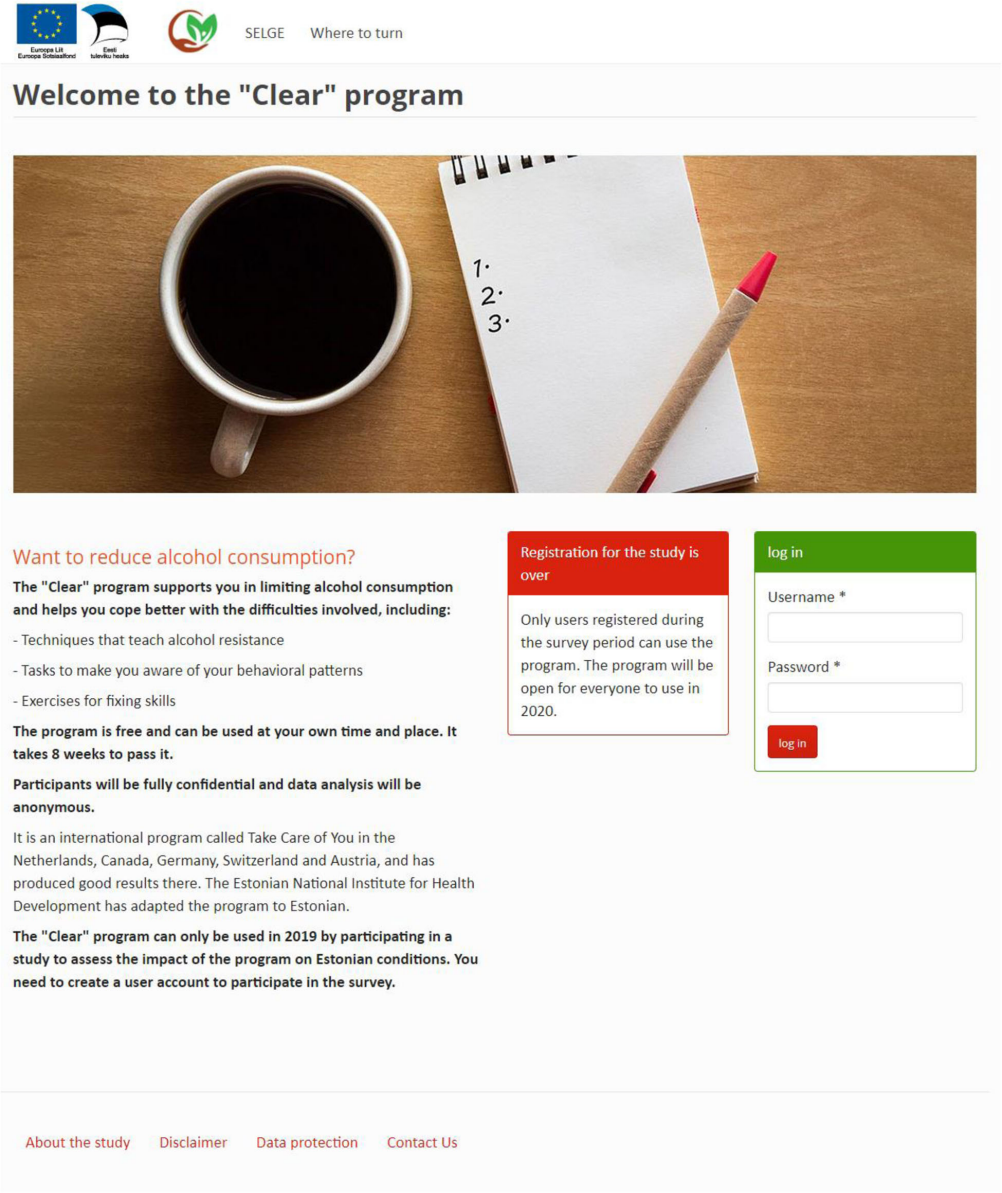
Web site landing page (translated from Estonian to English for publication purposes only)

Interested individuals are invited by a link to the study website (see Fig. 1). The corresponding landing page includes all pertinent study information and an informed consent page. Before consenting, individuals are told: 1) the inclusion/exclusion criteria of the study; 2) the potential risks/benefits of completing the intervention; 3) safety arrangements for during and after the study; 4) that participation is voluntary; and 5) that the study has been approved by the Tallinn Medical Research Ethics Committee. Participants are instructed in how to create a user account the *“Selge” program* and provided contacts to researchers for if they have any trouble. Informed consent is obtained by having individuals check several boxes, confirming that they have read and understood the terms of research. They also are asked for their telephone number, so we can call them for follow-up in case they ignore the prompts delivered by e-mail. To register an account, they need to create a username and indicate their email address. An automatic email with a confirmation link completes the registration process, after which the baseline assessment starts.

### Randomization and trial flow

**Fig. 2:**
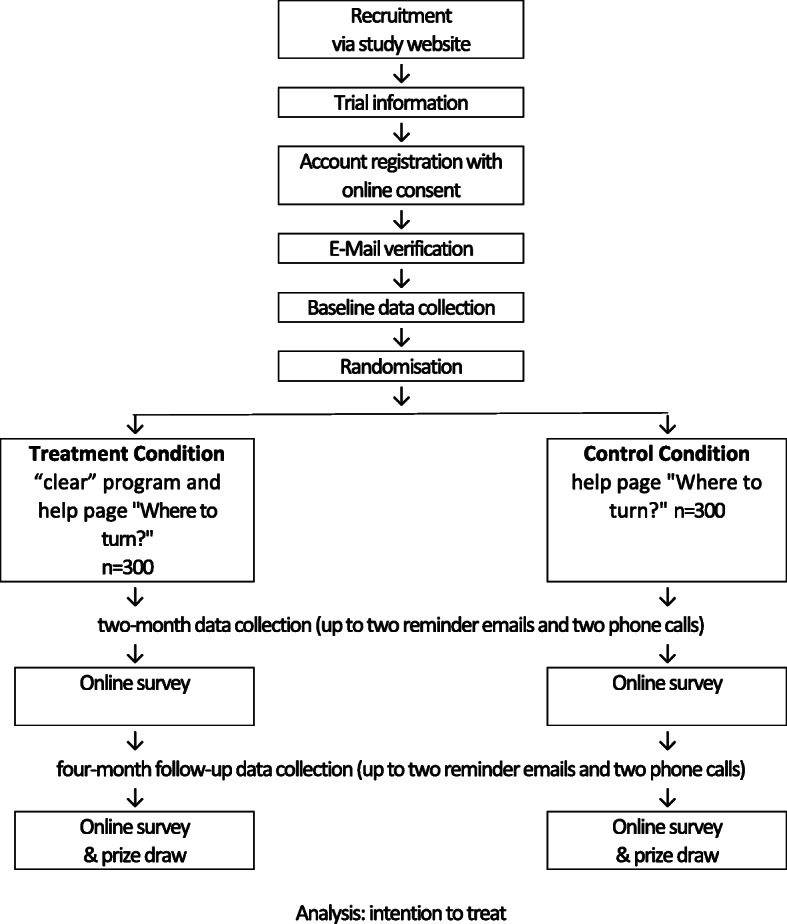
Randomization and trial flow chart

The baseline assessment starts with a suicidality screener [13]. Users indicating a higher risk of suicidality are excluded from the study and program and provided with a list of help lines. Users who do not meet the requirements for AUDIT are excluded from the study, but remain free to use the program, nevertheless. Users who fulfill the requirements after the baseline assessment are considered study participants and randomly assigned by a simple server randomization in a 1:1 ratio to either the *“Selge” program* or the control condition (see Fig. 2), informed about their assignment, and given further instructions. Participants in the control group are informed that they can use the program after 6 months, and provided with a help page — “Where to turn” — which is described in detail later in this paper. After 8 weeks (end of treatment), both groups undergo an assessment, as well as 6 months after baseline. An email prompt is sent automatically for each assessment, followed by two additional reminders sent to those who still have not completed the assessment. The last reminder sends a system email to the non-blinded study coordinator who then tries to contact the participant by phone up to twice. Participants are not compensated for completing the follow-up assessments. However, from all participants who complete all assessments, ten will be randomly selected to win a smartphone.

### Intervention and control condition

#### Treatment condition (“Selge” program)

The treatment website encompasses a dashboard, a drinking diary, 10 treatment modules and the help page “Where to turn?” (see control condition).

##### Modules

**Fig. 3:**
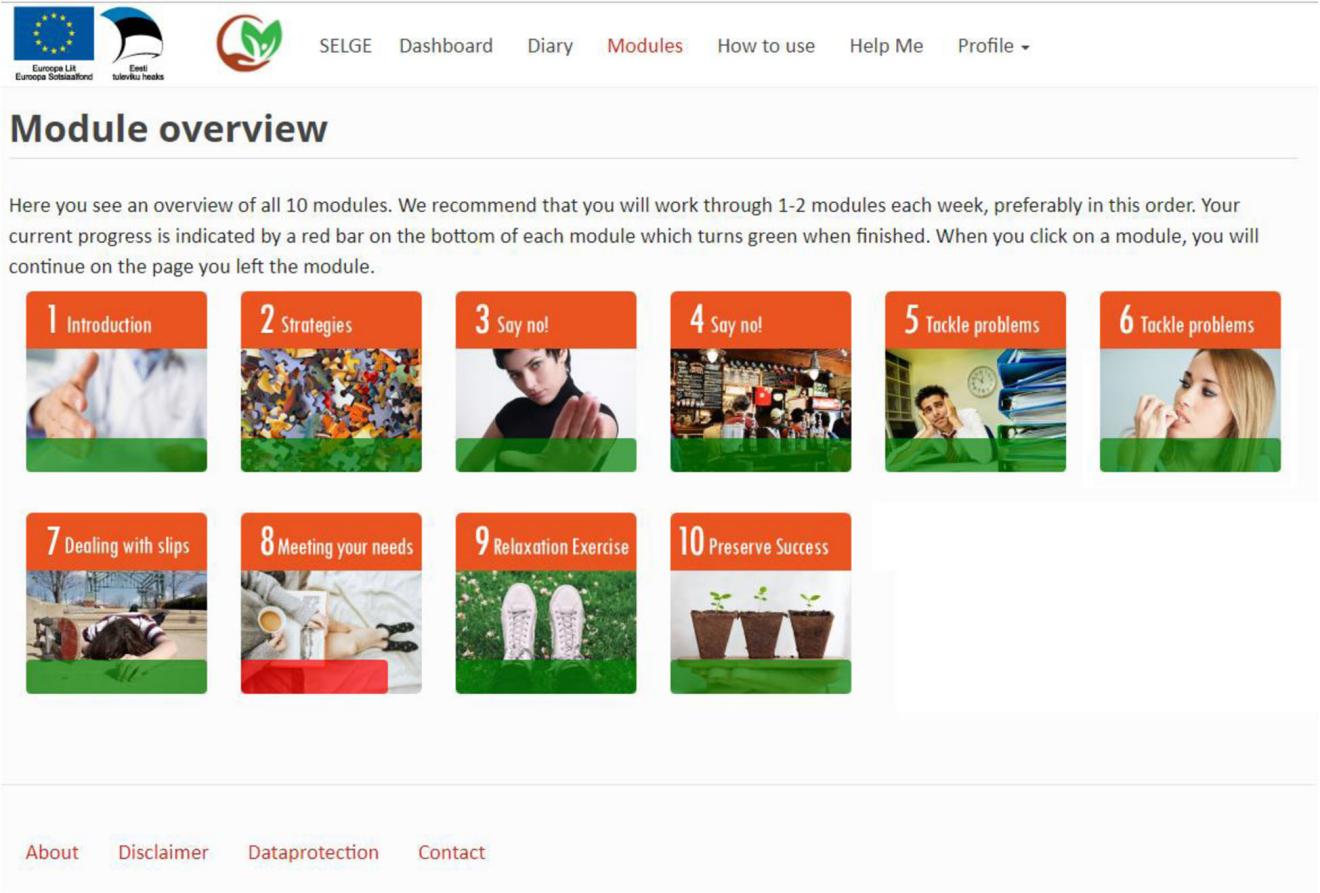
Main menu of intervention modules (translated from Estonian to English for publication purposes only)

Ten treatment modules (see Fig. 3) comprise the majority of this 8-week program. Participants are advised to complete the modules at their own pace, but in the order listed. However, they may freely skip or repeat any module, if they choose. The content of each module was created based upon the principles of CBT and MI (Table 1). The emphasis is on strategies to reduce negative thinking and worry, on behavioural activation, and on self-care (e.g., sleep hygiene). A progress bar, also visible on the dashboard, indicates each user’s progress in the module, which is saved page by page.

**Table 1:** Overview of Module Content

Module Number and Title	Module Content
M1: Introduction	• Introduction to the intervention • Motivational enhancement (i.e., identifying reasons for change, and pros and cons of drinking and not drinking) • Self-monitoring alcohol use and mood
M2: Strategies for Meeting Your Goals	• Strategies to change drinking habits, including stimulus control (e.g., ridding home of cues) • Resisting alcohol in specific situations (e.g., situations involving negative emotions) • Practicing refusal skills in high-risk situations • Developing personal strategies to reduce/abstain from harmful alcohol use
M3: Learning to “Say No” to Alcohol	• Learning about the various ways to say no to alcohol. • Ways to resist social pressures to drink • Trying out a few role-plays to practice drinking-refusal skills
M4: Identifying Risky Situations	• Identifying personal high-risk situations for drinking • Learning about “seemingly unimportant decisions” that could lead to heavy drinking
M5: Problem Solving	• Relating mood/alcohol issues to problems • The difference between controllable and uncontrollable problems • Formal problem solving (including problem identification, generating possible solutions, evaluating possible solutions, selecting a course of action, and evaluating the implementation of an action plan)
M6: Coping with Craving	• Psychoeducation about craving (e.g., different forms of craving [mental and physical]) • Introduce self-monitoring of craving • New ways to effectively cope with cravings (e.g., distraction, talking, experiencing the craving, and recalling the negative outcomes of drinking)
M7: Dealing with Slips	• Define a “slip” versus a full-blown relapse • Introduce ways to cope with slips in mood and/or drinking
M8: Meeting Your Needs	• “Sleep Hygiene” • Ruminating and worrying less • Increasing and/or improving social network
M9: Relaxation Exercise	• Key relaxation exercises to reduce stress (i.e., progressive muscle relaxation) • Deep breathing • Scheduling relaxation times during the week
M10: Preserve Your Success	• Identify “early warning signs” for slip/relapse • Create a personalized relapse-prevention plan • Discuss ways to cope with relapse • Identify top five coping strategies

##### Dashboard

The dashboard is the homepage after login and aims to provide a quick overview about the most important components of the program. Users see automated messages from their e-coach (see below), study-related information, module progress, and the last 2 weeks of their drinking diary. They can also plan some activities unrelated to alcohol use and upload a photo reminding them of this activity.

##### E-coach

The e-coach is, on one hand, a real person who can be contacted by e-mail and provide individual guidance. On the other hand, it is also an automated agent that sends e-mails and provides suggestions on the dashboard (e.g., to work through a module or to fill out an assessment if the time has come). One of the purposes of the e-coach is to help reduce attrition.

##### Social presence

Apart from the e-coach, a strong element of social presence is created via a brief introduction video at the start of each module. The actor seen in those videos is the same as the one depicted on the dashboard as the e-coach representing the *“Selge”* program team. Research suggests that such social factors may improve accountability [14], especially for self-guided programmes. Another social element is six fictional companions, one of whom each participant can select at the start of the program; someone they feel they might identify with, in terms of gender, age, occupation, and/or family background. These companions provide advice and examples at specific points during the modules.

##### Consumption diary

Participants in the intervention group will be asked to track their weekly alcohol use and goals measured in standard drinks throughout the program. Daily goals and actual intake will be displayed on a progression graph with two separate lines, making any deviation of intake from their target evident. A separate online drinking calculator [15] helps users to estimate the number of standard drinks they have consumed, by showing country-specific drinking containers/glasses/bottles, their different volumes in milliliters (ml), alcohol percentages and calories. Next to the calculator results, participants are reminded of their country-specific safe-drinking limits.

##### Control condition

**Fig. 4:**
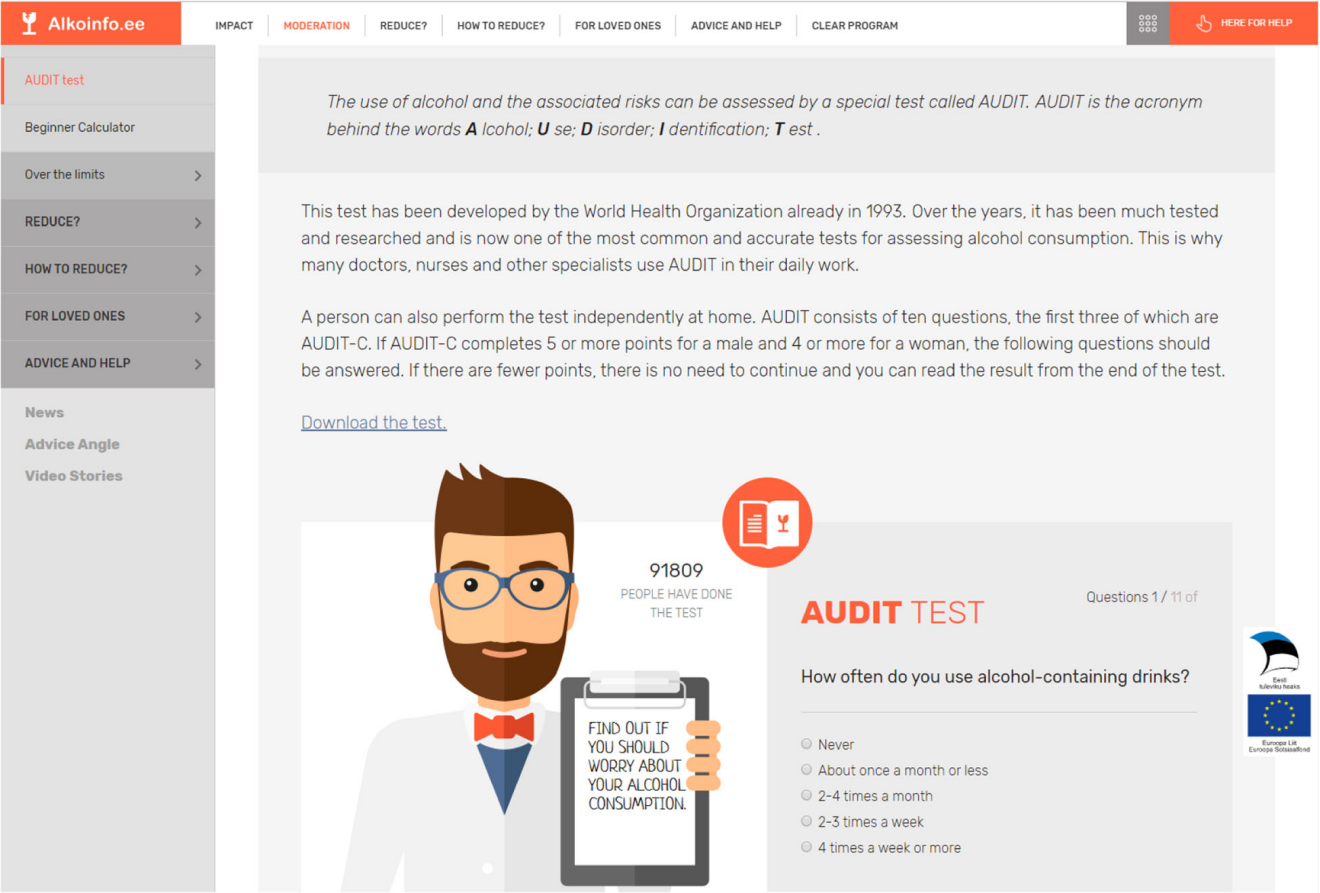
Self test for alcohol abuse (AUDIT) on alkoinfo.ee (translated from Estonian to English for publication purposes only)

Individuals assigned to the control condition are told that they will have access to the complete program in 6 months. In the interim, they only have access to a help page called “Where to turn”, which lists treatment centers (including contact details), psychiatric care providers and further health services. Moreover, they are advised to visit the website www.alkoinfo.ee [16], where they can learn about the principles of alcohol abuse treatment, complete a self-administered test based on the AUDIT [6] (see Fig. 4), and receive personalized feedback.

##### Technical specifications

The program website [17] is based on the content management system Drupal 7 and has a responsive design, making it suitable for both desktop computers and mobile devices. All connections from participants and administrators will run SSL-encrypted and password-protected. At any time, any participant will have access to his or her own data only. There are three administrative roles in place: (1) E-coaches have access to some of the participants’ data, like the diary or texts that participants enter during exercises in some modules; this information can assist their coaching activities. (2) Researchers have access to all data from questionnaires and the diary. (3) The administrator has full access to all data, including some technical meta data. All data that participants enter will undergo some input validation, ensuring a certain degree of data quality.

All data will be stored on webservers hosted by TAI at a research institution fileserver catalogue that is accessible to only a limited number of research project members. Data will be exported via Drupal and stored at the principal investigator’s institution on local computers for further processing and local fileservers for archiving. After the study ends, data sets will be stripped of all email addresses and phone numbers. The investigator affirms and upholds the principle of every participant’s right to privacy and ensures that all personnel involved in the study will comply with applicable privacy laws. No subject-identifying data will ever be published or presented at conferences.

#### Measures

Table 2 provides a schedule for each assessment measure. Demographic information will include age, gender, nationality, type of settlement (which major city or rural settlement), educational level, and the type of social engagement (employer, employed, student, retired, unemployed). All self-report questionnaires will be administered online.

**Table 2:** Schedule of Assessment Measures

Self-Report Measures	Baseline (T0)	8 weeks (T1)	6 months (T2)
1. Demographics	x		
2. Suicidality Screener (P4)	x		
3. Alcohol Use Disorder Identification Test (AUDIT, main outcome)	x	x	x
4. Self-reported number of standard drinks on typical week over the past 6 months	x	x	x
5. Self-reported number of alcohol-free days on a typical week over the past 6 months	x	x	x
6. Drinking Motives Questionnaire (DMQ-R-5)	x	x	x
7. Motivation for Change	x	x	x
8. Mental Health Inventory (MHI-5)	x	x	x
9. Drug Use (NIDA ASSIST)	x	x	x
10. Customer satisfaction (iCSQ-8)		x	
11. Usage of other resources for intervention or treatment of alcohol use disorders		x	x

##### Primary outcome

The primary outcome will be the change in the Alcohol Use Disorders Identification Test (AUDIT [6]) score between the 6-month follow-up and baseline assessments. The AUDIT is a 10-item self-report measure designed to assess alcohol misuse. Overall, the measure provides an indication of both alcohol use and alcohol-related problems. The AUDIT is a widely used, reliable, and validated estimate of alcohol misuse [6].

##### Secondary outcomes

Secondary outcomes include:

1. The self-reported number of (Estonian) standard drinks and alcohol-free days on a typical week over the past 6 months. This measure has been selected for use to allow for comparisons with results from an ongoing study being spear-headed by the World Health Organization [18].
2. Drinking motives are being measured using the Drinking Motives Questionnaire (DMQ-R-5). The DMQ-R measures how people score on four motivational dimensions. Originally developed by Cooper [19], the underlying factor structure was confirmed by Kuntsche et al. [20] in Switzerland. The four motive types are social (e.g., drinking to be sociable, to celebrate, at parties); coping (e.g., drinking because it makes you forget about problems); enhancement (e.g., drinking to feel better or to be able to do things otherwise impossible); and social pressure and conformity (e.g., drinking because others do, to fit in).
3. Motivation for Change (3 items). Participants rate how *important*, how *confident* and how *ready* they are to change their alcohol use, on scales from 0 to 10.
4. Mental health is being measured with the 5-item version of the Mental Health Inventory [21]. The MHI-5 is about recent mental distress and a self-reported diagnosis of depression. It is well validated and easy to use.
5. Treatment retention is defined as the percentage of data fields filled out in the weekly alcohol consumption diary over the 8 weeks of the intervention.
6. Further illicit drug consumption is being accounted for via the NIDA-Modified ASSIST questionnaire [22].
7. The “Customer Satisfaction Questionnaire” for internet interventions (iCSQ-8 [23]) is being used to assess satisfaction with the program in the intervention group. The iCSQ-8 has been extensively studied and results in scores from 8 to 32 (higher = better). We will implement a slightly-adapted version to account for Estonian practices.
8. To check if participants used the resources provided by the program (treatment group only) and/or if they used additional resources (both groups) to treat themselves for their alcohol use, they are asked how many hours they invested in such material. Moreover, they are asked whether they employed professional help (consultation) to reduce their alcohol use (GP, psychologist, psychiatrist, other health specialist); and, if so, how many hours.

### Sample size calculation

A recent meta-analysis [24] showed that combining CBT and MI resulted in statistically-significant reductions in alcohol use, with effect sizes in the small range for internet interventions (*g* = .20). As we aim to compare the *“Selge” program* with a control condition which provides extensive resources and a self-administered test of alcohol abuse, we expect larger effects. The study that comes closest to our design compared online Personalized Normative Feedback (PNF) plus an intensive, but unguided CBT (56 daily sessions plus six weekly follow up sessions) program versus PNF with an online booklet; it also recruited alcohol misusers from the general population and it generated a Cohens’ *d* = 0.20 [11]. As we are offering a minimal-guidance intervention and comparing it to a self-administered test with routine advice on usual treatment, we expect a Cohens’ *d* = 0.23 for the current study. Using G*Power, the sample size required to detect this small effect with 80% power and α = 0.05 is *N* = 596 total (298 per group).

### Data analysis plan

All analyses will be guided by the intention to treat (ITT) principle. Preliminary testing will assess two things: differences between the two study arms at baseline; and the degree of missing data. Baseline differences will be analysed by (generalised) linear mixed models (LMM). Missing data will be addressed by multiple imputation procedures from Amelia II through R [25]. Imputed data will be analysed with multinomial and binary regression models, considering two-sided tests with a type I error rate of *p* < 0.05 as statistically significant. Results will be cross-checked with complete case analyses of non-imputed data. Besides ITT analyses, we will also perform per-protocol analyses.

## Discussion

The planned study will evaluate the efficacy and the potential for referral to treatment as usual of the self-help *“Selge” program* for alcohol misusers and those with likely alcohol dependence in Estonia. This will be the first internet program provided in Estonia with culturally-adapted contents and state-of-the-art design and technology, and will be directly implemented in a larger, country-wide health promotion campaign. This comprises a unique opportunity, from both an intervention and research perspective. Our goals are to achieve maximum attention in the general population of Estonia and reach as many individuals from the target group as possible, similar to a recent screening and brief intervention Website [7] – with most of those in the target population having never received any prior alcohol treatment at all. Most similar programs that were introduce in other countries have had to compete with other already-existing internet programs for the reduction of alcohol misuse, among which most likely had never been evaluated for efficacy, and most lacked evidence-based therapeutic content [26]. Thus, we expect somewhat higher intervention adherence as observed in similar studies in other countries [10, 12]. This is, amongst others, one reason why we decided to have an active control group consisting of a self-administered test as well as advice on and how to access treatment as usual. Such a direct comparison has only been reported for one other study [11], which contained several methodological limitations. If the *“Selge” program* turns out to be effective, it will be continuously advertised and implemented as an important part of the continuum of intervention and care for alcohol misuse and dependence in Estonia. Moreover, maintenance of the program would be guaranteed for system and content updates in the future, a feature that is rarely the case for internet interventions for the reduction of alcohol misuse, due to the inherent limitations of research funds [27].

We chose not to limit our program to harmful and hazardous alcohol users only (AUDIT scores ≥8 and < 20), but also to include alcohol users suggestive of dependence for two reasons. First, we did not want to limit access to the intervention since this is the first program in Estonia of its kind and treatment coverage remains low [5]. Second, we wanted to allow for comparison of our results against other studies, like the one spear-headed by the World Health Organization (WHO) [18].

We have decided to conduct both a post-intervention measurement and a follow-up measurement in order to distinguish the intervention effects from any follow-up effects. However, this can reduce the post-intervention and follow-up survey rates, especially since it is an internet intervention. To minimize the risk of excessive loss to follow-up, we have integrated into our design additional telephone calls. We therefore expect follow-up rates of 50–70% at post-intervention and 40–60% at 6 months follow-up [10, 12].

As with every internet-based program for the reduction of alcohol use, we expect a comparatively-high rate of dropout. However, due to the central implementation of the program in Estonians’ alcohol intervention efforts, its uniqueness in Estonia, and its cultural adaptations which include videos in each module, we expect that treatment adherence will be relatively high and the dropout rate relatively low.

Primary trial findings will be published in open-access journals, so both the public and policymakers in Estonia can learn about the intervention and the study results on 2020. Participant-friendly summaries of trial findings will be published on the program website.

## Data Availability

Anonymized study data will be available upon request. Participant-friendly summaries of trial findings will be published on the program website.

## Abbreviations

RCT: Randomized controlled trial
CBT: Cognitive behavioural therapy
MI: Motivational interviewing
TLFB: Timeline Follow Back
MHI-5: Short version of the Mental Health Inventory
AUDIT: Alcohol Use Disorder Identification Test
NIDA-ASSIST: National Institute on Drug Abuse, Alcohol, Smoking, and Substance Involvement Screening Test
TAI: Tervise Arengu Instituut (Estonian National Institute for Health Development)
PNF: Personalized Normative Feedback
ITT: Intention to treat
GP: General Practitioner
